# To the moon: Retail investor attention and sentiment across asset types in online media

**DOI:** 10.1371/journal.pone.0349616

**Published:** 2026-06-10

**Authors:** Michael L. Smith, Nicole J. Olynk Widmar, Valerie Kilders

**Affiliations:** Purdue University, Department of Agricultural Economics, West Lafayette, Indiana, United States of America; Indian Institute of Management Sambalpur, INDIA

## Abstract

Social media facilitates community building for users of varied and broad interests. Personal and retail (individual non-professional) investment communities have grown popular, and some engage in commentary on currencies and market events, while others coordinate action within markets. This analysis quantifies online and social media data about the US dollar and gold, as well as cryptocurrencies and stocks subject to attention shocks (including meme stocks), from 2020 through 2023. Using Quid’s social media listening platform, mentions of these four distinct topics were collected and analyzed separately to quantify the volume of mentions and sentiment of search results on a weekly basis. Statistical relationships between the asset groups and major events in the study period are explored; we assess public reactions to the regional bank failures, GameStop and AMC short squeeze events and the evolution of the crypto market. Large volumes of online media commentary on these topics suggest a sustained high level of interest in currency and investment markets, with evidence of the increasing influence of online communities in how the public views or talks about these markets.

## 1 Introduction

The US financial system is comprised of a diverse array of institutions and markets [1]. Investor sentiment is highly consequential and can have a virtuous relationship with policies and decisions of large institutions [2–4]. Studies of drivers of financial participation find what many financial professionals maintain: sentiment, including trust, is a key driver of participation in the financial system [5–7] and as such can be useful to developing competitive advantages [8]. Furthermore, investor sentiment has been found to have a positive and significant influence on cryptocurrency and stock returns in recent years [4].

Recent statistics show a decline of public trust in financial systems, with the share of individuals trusting financial institutions dropping from 28% in 2021 to only 20% in 2023 [9]. This decline in trust is critical given that it is a fundamental tenant of our financial systems [10]. The decline in trust is fueled by instances of financial recession and bank failure, which worsen banks’ reputation [11,12]. Notably, during the week of March 10^th^, 2023, Silicon Valley Bank (a regional bank in the US) collapsed [13] triggering runs on other regional banks (Signiture, which closed on March 12^th^, 2023, as well as First Republic and Silvergate which failed later) [14].

Alongside these instances, questions have started to arise about the stability of the United States Dollar (USD), a currency that has long been the preferred reserve currency due to its stability, strong growth in the US economy, and the democratic nature of the US system of governance [15–17]. Gold, meanwhile, has remained historically important, which is partially attributable to the gold standard (which is now defunct) and continues to be a highly regarded and popularly traded investment of interest [18].

However, while the US public debates trust in, and future stability of, the longstanding currency and precious metal markets, notably different – and highly volatile – markets have seen significant activity [19,20]. One example of this trend is found in the trading of cryptocurrencies and stocks that see rapid attention shocks in online media, which we will refer to as attention stocks. We define attention stocks as equities, whose trading activity and volatility are strongly influenced by abrupt shifts in online and social media attention. This includes, but is not limited to so called meme stocks, which are defined as extremely volatile stocks driven by online interest and social media hype leading retail investors to believe they can make money, instead of investors being attracted by company fundamentals [21]. Meme stocks in online and social media are is increasingly well studied [22–24]. Yet, most existing studies focus on singular attention stocks or meme stocks in isolation or as a class of assets with no contrast to more traditional assets such as the United States Dollar (USD) and gold.

A crucial factor in the proliferation of investments in such volatile and untraditional assets is consumer’s ability to exchange information in online spaces. The internet has changed how consumers shop and invest by lowering the cost of marketing for producers and reducing the cost of finding information for consumers including information for investors [25,26]. Research on the diffusion of information in trading markets suggests that the fluctuation in beliefs and expectations produces trading volume [27] creating financial ripple effects beyond the online communities, where users act as both producers and consumers of information [28].

Correspondingly, internet communities have emerged, centered around retail investors (who operate as individuals and are not professionals focused on long term growth) and day traders (who buy and sell within the same day) who comment, call to action, and report on financial matters [29,30]. Peer reviewed studies show that many of these individuals are not only motivated by profit but also by pleasure in the process that accompanies investing [31]. Indeed, while social media users increasingly end up in networks curated to them with like-minded individuals [32], researchers and political leaders alike warn about the effects of these social media networks, which can become isolated, bias-reinforcing echo chambers [32].

Building on the existing literature and the identified gaps, the central research question of this paper is as follows: how do public interest and sentiment expressed in online media differ across traditional and non-traditional asset types and evolve over time when measured using a consistent data collection and analytical framework? To address this research question, this paper employs online and social media listening to examine online media about attention stocks, cryptocurrencies, gold markets, and the USD. Each of these four topics is analyzed individually to discern the key events and their impact on social media interactions (mention count and net sentiment). Yet, critically, we use a consistent data collection process and methodological framework making results comparable across assets. For a more structured and in-depth analysis we subdivide each of these four topics into 3 thematic sub-searches studying commentary on *market institutions, market turbulence, and market choices*. These thematic sub-searches permit us to pinpoint when social media discussions focus on buy/sell debates (sub-search for *choices),* events including recession or inflation (*turbulence*), and the institutions that govern and run many of these markets (*institutions*).

With this consistent sampling strategy applied across our four distinct asset types, we generate comparative insights into how different investments are discussed online and how public interest and sentiment vary across markets and over time. Addressing this question is particularly relevant given the growing prominence of retail investor communities, the increasing institutionalization of cryptocurrencies, and renewed debate about trust in traditional financial markets. As such, these insights help academics and industry investment managers alike to better understand the emotional framing that drives public interest in these four distinct topics.

### 1.1 Background on currencies and related markets studied

Our paper focuses on four different investment groups. USD and gold were selected as traditionally steady investments or legal tender [16, 33], while attention stocks and cryptos represent more recently emerged alternative investment tools. The following sections provide some additional context for both groups.

#### Traditional financial markets.

The USD has been the defacto global reserve currency for decades and has remained resilient through recent events that brought turmoil to US and international markets including the 2008 financial crisis, the 2020 COVID pandemic, and subsequent global inflation [34]. Being the global reserve currency, the USD maintains a central role in the functioning of international economic systems [35]. Yet, pockets of distrust in the USD persist in the US, fueled by consumer price inflation and the use of economic sanctions [36].

Historically, worsening inflation has been linked to the public’s renewed interest in a return to the gold standard [37,38]. Gold has an ancient tradition of use as a symbolic material, a medium of exchange, and a value holder [39]. The USD was originally backed by gold and silver [40], but ultimately left the gold standard in 1971, transitioning to a floating currency [41]. Since 1971, consumer interest in returning to the gold standard has waxed and waned in the US, with some economists suggesting this correlates with a rise in inflation [40]. Despite no longer serving as backing for the USD, gold remains a prominent commodity.

#### Emerging and alternative investment tools.

In recent years, investors and regular consumers have increasingly focused on cryptocurrencies with an estimated market size of $5.7 billion in 2024 [42]. Cryptocurrencies are digital currencies that are traditionally not regulated through governments or banks, but rather work through computer networks. In October 2021, the US SEC approved the first trading fund for a cryptocurrency [43,44].

Cryptocurrencies were initially styled as an alternative to fiat currencies [45]. They were supposed to be easy-to-use mediums of exchange, untraceable, and unable to be diluted by inflation or a central bank [46,47]. However, these attributes have been increasingly called into question. Some experts do not see cryptocurrencies as an effective inflation hedge [47]. Furthermore, the myth of the privacy of these cryptocurrencies, namely bitcoin, has long been questioned and disputed by experts, and more recently demonstrated to be false by law enforcement [46, 48, 49].

Also associated with a substantial level of risk, the concept of attention stocks has risen to popularity in recent years especially among retail investors that are active in the online space [50]. A critical example is the January 2021 GameStop and AMC short squeeze, in which individual investors exploited the positions of institutional investors [51]. A financial short squeeze is an action to place a bet that a stock will fall; as the value declines, the investor’s portfolio generates a profit [52]. Both companies had long-term business issues due to the evolution in consumer preferences, and short-term issues generated by the COVID-19 pandemic. GameStop and AMC faced headwinds in a market that is increasingly digital, reducing the need for in-person stores [53,54]. In early 2021, retail investors on forums like Reddit’s r/WallStreetBets coordinated to buy heavily shorted stocks such as GameStop (GME) and AMC, driving up their prices and forcing hedge funds with large short positions to cover their bets at huge losses [55], all the while generating extreme price volatility [56,57]. The positive shock in their stock prices did not relate to any change in the fundamentals that underpin the stock value (such as macroeconomics, financial statements, and indicators of intrinsic value [58]), nor was this the result of new information to investors [22,59]. Research suggests that this resembles a coordinated effort to “pump and dump” or manipulate the market with the intention to make a quick financial gain, rather than to hold these assets for a long time [22,60]. Those who delivered initial calls to action had much to gain from the short squeeze event.

With both AMC and GameStop, market manipulation (in favor of increasing the stock price) precedes events favorable to the companies’ financial health. Both companies responded to the events by issuing shares and paying down a combined roughly $800 million in debt [61]. The Federal Reserve concluded that this is a model of self-fulfilling expectations on the part of investors [62].

The attention stocks category also included a stock that was affected by investor activism but in a contrasting way, which is included as a counterfactual: Bud Light. While both AMC and GameStop can be classified as meme stocks, Bud Light is not a meme stock. It did, however, face a brand crisis caused by dissatisfaction experienced by some of its customer base resulting in substantial stock volatility and downward pressure on stock price. Much of this discourse, including a 2023 boycott and calls to action to sell the stock, occurred on social media. These centered around certain groups protesting an actor included in one of their advertisements [63], qualifying it as an attention stock. The boycott, a response to a company attempt to grow the business by changing brand perceptions, had immediate effects. The firm saw sales and stock price fall by roughly 20% in just a few weeks [63,64]. While these online media users had different motivations than those that impacted AMC and GameStop, all three of these assets were subject to the stock volatility craze, wherein online-based interest and sentiments were successful in driving price and trading activity [55,65]. As such, they allow us to compare differences within attention stocks with regards to attention and sentiment.

## 2 Materials and methods

To assess online chatter about the four investment groups, we leverage social media listening. Online and social media listening has emerged as a tool for researchers to better understand the interests of, opinions on, and sentiment about various topics by a population. Social media listening allows researchers to collect data from members of the public without prompting them, reducing biases that may emerge in a survey [66]. Online and social media listening also offers researchers an opportunity to examine the volume of conversation about a subject, which may vary over time, and to see how social media users’ feelings (or sentiments) evolve, including in their reaction to real-life real-time events [67,68]. Social media listening generally uses techniques of searching/finding soundbites from social media posts that mention specific keyword terms.

We use data collected from online and social media platforms through an online and social listening platform called Quid (formerly known as Netbase) [69]. Quid has been used by researchers to study a multitude of topics relevant to investments, including stock price movements for entertainment companies [66,70] and stock prices for cruise companies facing pandemic-era disruptions [71]. Using Quid, researchers organize a search topic using keywords, date ranges, and geographic or other filters to collect search results. We use the Quid platform to collect publicly available data from third-party sites (news and social media providers) in compliance with the usage terms for these sites; Quid does not collect individually identifiable information [72,73]. We only collect posts that originate in the US, and we (the researchers) do not filter out suspected bot accounts.

The topics developed for data collection and analysis were our four investment groups: USD, gold, stocks (AMC,GME, and BUD), and cryptocurrencies. While countless cryptocurrencies and tokens exist, we only focus Bitcoin (BTC) and Ethereum (ETH), which are the leading cryptocurrencies [74]. Interest in these four distinct topics is very high, which generates a limitation in the amount of assets we are able to study. This is most clearly seen in the Crypto and Attention Stocks search. Inevitably, we encounter a tradeoff between breath of assets studied, at the expense of the depth of sampling into conversations about each of these. This analysis focuses deeply on a few illustrative examples of each topic, rather than incorporating an exhaustive list of possible assets under each topic.

The search terms used in each of the four distinct topical searches are described in Table 1. Online and social media posts originating in the US that contain these primary search terms were included in data collection. Due to the nature of online data, issues like slang, expressions, and homonyms may exist wherein a post is included due to the mention of one or more primary terms, but the post itself may not actually relate to the topic of the search. Exclusionary terms help us to remove unrelated media from search results. The number of mentions is greater than the number of posts because a single post might contain multiple mentions [71].

**Table 1:** Primary Inclusionary Search Terms for Online and Social Media Searches.

Gold	USD	Attention Stocks	Crypto
gold price	United States Dollar	AMC	Bitcoin
price of gold	united states dollar	#AMC	Ethereum
gold standard	federal reserve banknote	Gamestop	BTC
	American dollar	GME	ETH
	US dollar	#GME	#bitcoin
	dollar value	budlight	#BTC
	exchange rate	AB InBev	#ethereum
	strengthen the dollar	Anheuser-Busch	#ETH
	weaken the dollar	BUD	
	currency	#BUD	

We study posts and mentions originating in the US between January 1, 2020, and December 31, 2023. Data was collected February 5^th^, 2024, and February 9^th^, 2024. Collecting the dataset within a consistent and short period of time is important due to the nature of social media; a post may be deleted at any time, leading to it no longer being recorded in later data collection periods [67]. As such, collecting it in a brief period of time facilitates our comparative analysis across the asset types.

As mentioned, data is collected on each of the four topics (USD, Gold, attention stocks, and Cryptos) individually. Building on descriptive text analysis conducted by Fisk et al. [63], data collected is subdivided into 3 thematic sub-searches to study comments on *market institutions, market turbulence, and market choices*. In doing so, we examine specific discussions about buying/selling (sub-search for *choices),* market shocks like recession or inflation (*turbulence*), and the governing and operating institutions of these markets (*institutions*). Terms for each thematic sub-search are included in Table 2 (asset terms) and Table 3 (market terms). Terms excluded from the online media collection are detailed in the appendix (S1 Files see Appendix Excluded terms). Exclusionary terms in total reduced the number of mentions collected by roughly 15%. The S1 Files appendix also includes an extended table of descriptive statistics that breakdown mentions of each asset group including their sub-search sizes. The full dataset is available in S2 File Dataset.

**Table 2:** Asset Groups Sub-Search Terms.

AMC	Bitcoin	Bud Light	Ethereum	GameStop
AMC	Bitcoin	Bud Light	Ethereum	Gamestop
amc	BTC	Budlight	ETH	gamestop
#AMC	#bitcoin	budlight	#ethereum	gme
#amc	bitcoin	bud light	#eth	GME
	#btc	BUD	#ETH	#GME
		#BUD	eth	#gme
		bud		#gamestop
		#bud		#Gamestop
		AB inBEV		
		AB Inbev		
		Anheuser-Busch		
		AB InBev		

**Table 3:** Markets Sub-Search Inclusionary Terms.

	Institutions		Market Choices		Market Turbulence
balance sheet	Federal Reserve Chairman	quantitative easing	bull market	options market	recession
Ben Bernanke	Federal Reserve System	regulators	#FOMO	sell	#inflation
bernanke	FINANCIAL INDUSTRY REGULATORY AUTHORITY	reserve ratio	#FUD	short	#interestrates
Bernanke	FINRA	safe haven	#liquid	short sell	#recession
CFPB	FIO	SEC	#liquidity	to the moon	#softlanding
CFTC	gold standard	sec	#sellnow	volatile	basket of goods
chair of the fed	inflation	securities and exchange commission	#tothemoon	volatility	consumer inflation
chairman of the fed	inflationary	Securities and Exchange Commission	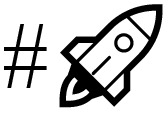	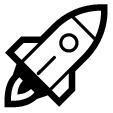	high price
Chairman of the Federal Reserve	Internal Revenue Service	Securities Investor Protection Corp.	bear market		high prices
Commodity Futures Trading Commission	IRS	SIPC	bearish		inflation
Consumer Financial Protection Bureau	Jerome Powell	the fed	bullish		inflation rate
deflation	macro	United States Department of Labor	buy		interest rates
deflationary	monetary	United States Department of the Treasury	day trade		inventory
dollar value	monetary economics	United States Secret Service	day trader		leading indicators
economist	National Credit Union Administration	US Department of Labor	fire sale		Recession
FBI	NCUA	US Department of the Treasury	FOMO		shocks
FDIC	OCC	US Department of Treasury	FUD		soft landing
fed chair	Office of the Comptroller of the Currency	USSS	liquid		stagflation
Federal Bureau of Investigation	Office of Thrift Supervision	value	liquidity		supply chain
Federal Deposit Insurance Corp.	OTS		margin trading		supply shortage
Federal Insurance Office	Powell		options		transitory
federal reserve	powell				unemployment
					yield curve

To better understand how online and social media authors talk about a subject, sentiment analysis is employed to examine the tone and context of a post. Posts (which contain one or more mentions) are analyzed through the natural language processor (NLP) provided by Quid Monitor. This NLP assigns each post a positive, negative, or neutral score [69]. Quid also reports a time-varying net sentiment score, which reflects the percent of positive minus the percent of negative posts [69]. This net sentiment score is bound to the range [−100, 100]. Due to the high-cost nature of natural language processing, net sentiment counts reflect a sample of 10% of posts collected, which are then scaled up to 100% to give an image of the total dataset [67]. The top terms used in online media posts are described in two contexts, “behavior” (verbal) terms and “attribute” (adjective) terms [69].

To better understand the differences in perceptions between total mention counts (and corresponding net sentiment) of our four groups (USD, gold, attention stocks, and cryptocurrencies) we employ a T-test of means and a F-test of variance on the net sentiment between each asset group. To evaluate means, we employ the mean-comparison test with the null hypothesis that there is no difference in mean net sentiment between groups [75]. We compliment this with the classical F-test for variance to test for differences in variance between asset groups [76]. We employ these same tests to study net sentiment within our four asset groups before and after the regional bank collapses which occurred on and around March 10^th^, 2023, comparing net sentiment before that week with net sentiment after that week (omitting the week beginning March 5^th^, 2023).

## 3 Results and discussion

Descriptive statistics on weekly mentions of the four asset groups are described in Table 4. We begin by discussing these statistics with respect to each asset group as well as common terms used to determine sentiment scoring by the NLP.

**Table 4:** Average (Standard Deviation) of Weekly Net Sentiment and Mentions.

Values	2020	2021	2022	2023
Net Sentiment Gold	18.3	21.9	21.3	24.1
	(13.9)	(12.2)	(10.5)	(13.6)
Net Sentiment USD	12.2	17.8	14.3	3.9
	(9.1)	(11.3)	(13.0)	(16.3)
Net Sentiment Attention Stocks	43.2	46.7	46.2	18.7
	(18.5)	(9.8)	(11.1)	(28.6)
Net Sentiment Crypto	50.8	47.1	42.4	54.0
	(7.0)	(6.2)	(8.8)	(10.0)
Mentions Gold	8,799	8,497	9,267	11,961
	(2,819)	(1,665)	(2,187)	(3,442)
Mentions USD	109,822	252,035	224,712	213,650
	(16,628)	(70,381)	(43,964)	(55,914)
Mentions Attention Stocks	102,347	211,643	151,463	342,015
	(18,269)	(270,374)	(25,760)	(178, 098)
Mentions Crypto	433,965	1,086,673	1,173,269	2,429,134
	(138,369)	(153,623)	(155,523)	(2,238,917)

### 3.1 USD

**Fig 1:**
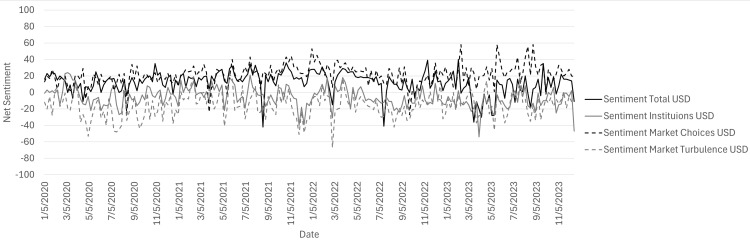
Net Sentiment of USD.

**Fig 2:**
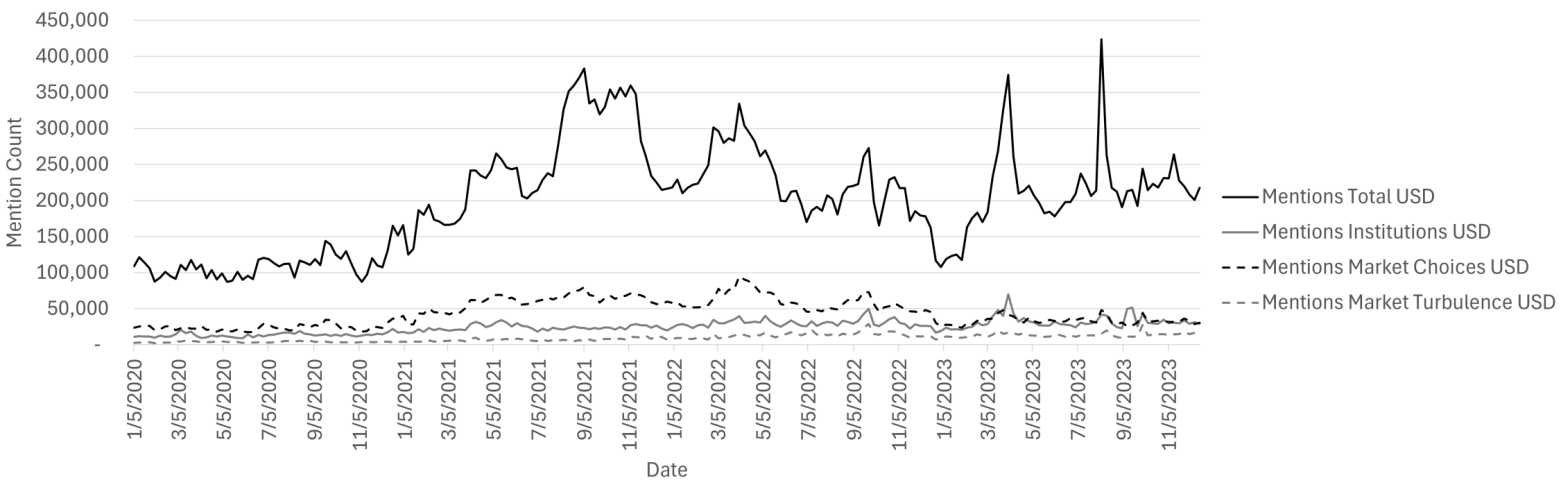
Mentions of USD.

Net Sentiment about the USD remains positive, albeit lower in absolute value than gold, attention stocks, or cryptocurrencies studied. Throughout the period of study, the USD experienced relatively little in terms of news or market happenings, compared to the other topics. However, over our focal period, volume of mentions in USD *turbulence* and USD *institutions* grew considerably. Fig 1 shows net sentiment of posts about USD and Fig 2 visualizes mention counts of USD.

Large spikes in activity were observed around August of 2021, as well as April and August 2023. In the latter half of 2021, the USD was trading against the Euro at the lowest it had been since 2018 [77], and social media commentators appear to have expected that the dollar would soon strengthen. Aligning with the spike in 2023, discussions emerged regarding governance of the USD discussing the aforementioned US House bill to return to a gold standard [78]. Additionally, a bill emerged in the State Legislature of Texas to create a gold and silver-backed currency as well as the establishment of a digital form of the currency which generated discussion online [79]. Both bills were ultimately unsuccessful. Simultaneously, a debate emerged regarding a digital currency from the Federal Reserve [80–82]. In late July 2023, CPI data was released which led to speculation that the FOMC would keep interest rates steady in response to persistent inflation, which generated discussion on social media into early August [83]. Indeed, the top terms captured during our observation period for USD reflects these debates as highlighted in Table 5. To ‘use’ and to ‘not use’ are common behavioral contexts for mentions, reflecting the pervasive usability of the Dollar. Also present are discussions of a virtual US currency, with over 11,000 mentions in 2020, growing to over 20,000 mentions in 2023. Interestingly, the USD top terms also include mentions of cryptocurrency, increasing from over 18,000 mentions in 2021 to over 35,000 mentions in 2023, with many of the dollar’s detractors singing praise to attention stocks, gold, and crypto.

**Table 5:** Leading Terms for USD and Gold in Social Media Data.

Category	USD Leading Terms				Gold Leading Terms			
	2020	2021	2022	2023	2020	2021	2022	2023
Positive Behavior #1	use	use	use	use	go back	go back	go back	go back
Positive Behavior #2	buy	buy	buy	buy	switch	use	use	use
Negative Behavior #1	not purchase	ban	not use	ban	abandon	abandon	abandon	abandon
Negative Behavior #2	not use	not purchase	not purchase	not use	not want	not use	weigh on	weigh on
Positive Attribute #1	virtual currency	crypto currency	crypto currency	crypto currency	current gold standard	fall	fall	fall
Positive Attribute #2	dollar	dollar	technological dimension	dollar	fall	gain	gain	gain
Negative Attribute #1	dollar	lose	dollar	dollar	rise	rise	rise	rise
Negative Attribute #2	lose	dollar	lose	lose	spot gold price	price	price	GBP/USD News Gold price

### 3.2 Gold

**Fig 3:**
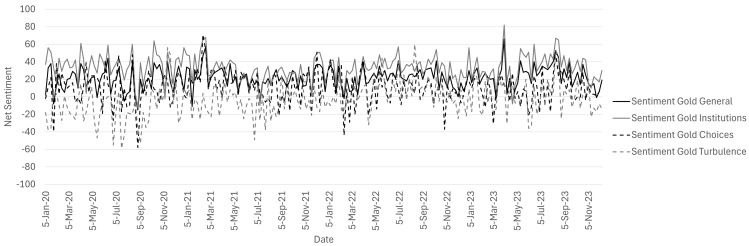
Net Sentiment of Gold.

**Fig 4:**
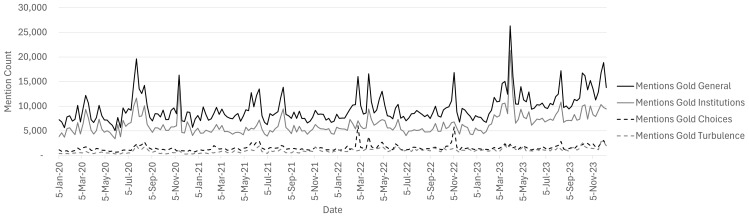
Mentions of Gold.

Gold weekly net sentiment, shown in Fig 3, remained mostly steady, with annual averages ranging around +20, it enjoyed a climb in net sentiment (specifically in the *institutions* theme) in early 2023. This may be related to the collapse of the Silicon Valley Bank, an event that market watchers suggested pushed investors toward gold markets [84]. Gold saw a climb in mentions, shown in Fig 4, throughout the study period.

This event is an opportunity for future research to examine the causal relationship between regional bank failures and public interest in gold. Recently, the acquisition of real gold bars has increased at retailers like Costco [85], indicating growing consumer interest in purchasing the physical product. It is worth noting that this type of physical acquisition is different from the digital forms that financial management and transactions tend to utilize in the 2020s [86].

The idea of gold as an investor’s safe haven is relatively old, but new research suggests investors’ appetite (and perceptions) for gold depends on market conditions [87]. These findings are largely corroborated by what we find. Commonly used attribute terms, described in Table 5, are oriented towards speculation. Mentions of prices ‘falling’ and ‘rising’ are frequent. We can see from social media data that discussions persist about a return to the gold standard. This discussion is largely among fringe communities. Nevertheless, in March of 2023, we also saw the proposal of H.R. 2435, the Gold Standard Restoration Act, which was referred to and died in the House Committee on Financial Services [78]. Like in the USD search results, social media commentators frequently discussed a return to the gold standard [37]. Notably, there is not much discussion about the reasons the US and other nations abandoned the gold standard, as suggested by earlier studies [38]. Instead, we find that “Go back”, “switch”, and “Abandon” are commonly used phrases in posts mentioning gold, usually referring to a return to (or abandonment of) the gold standard.

### 3.3 Attention stocks

**Fig 5:**
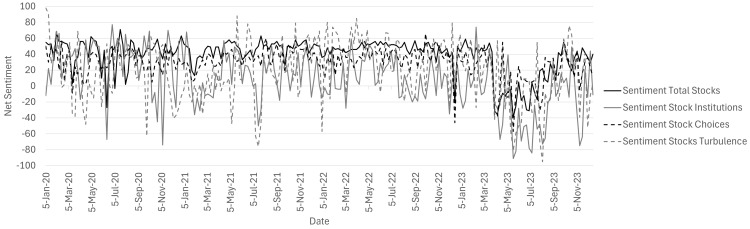
Net Sentiment of Attention Stocks.

**Fig 6:**
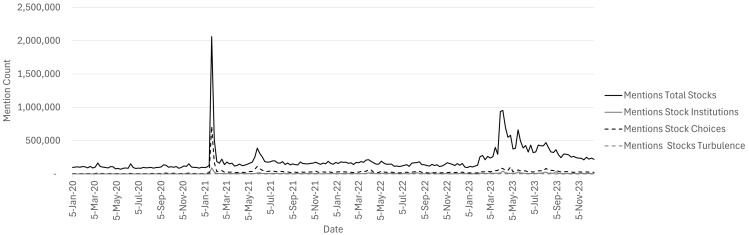
Mentions of Attention Stocks.

Online media for attention stocks, shown in Fig 5 (net sentiment) and Fig 6 (mentions) enjoyed a strong and positive weekly net sentiment of around +45 for the first 3 years, on average, but tapering off in 2023 (falling to +19). One notable deviation from this otherwise positive net sentiment occurred during the COVID-19 lockdowns. More specifically, the week of March 15^th^, 2020, saw net sentiment fall 40 points to +3, before quickly rebounding to +32 the next week. This 2023 shift coincides with mentions of Bud Light tripling in 2023 (from an average of 65,000/week to over 235,000/week in 2023).

GameStop and AMC saw their highest level of mentions in 2021: 80,000/week and 88,000/week, respectively. This represents a significant increase compared to the year prior when both averaged 16,000 mentions/week. During this period, average net sentiment rose modestly for each, ranging from +20 to +35 weekly average in 2021, respectively. For context, events leading up to the short squeeze were not favorable to business health for these companies. COVID-19 created issues for both GameStop and AMC and particularly the latter struggled in 2020, due to restrictions on public gatherings [88]. AMC suffered a 5-month hiatus without operation during the spring and summer of 2020, eventually reopening in August 2020 [89]. GameStop was reluctant to close, instead declaring it would remain open, inviting consternation on social media and in the news [90]. After some backlash, GameStop closed its doors to walk-in customers but remained open to curbside and online offerings [91]. AMC attracted attention by reversing its stance on masking and theater reopening, having first declared that masks would not be required once theaters reopened, but ultimately issuing a requirement [92].

We observe further declining net sentiment of attention stocks when searching for mentions of institutions beginning in 2023 (the 2023 weekly average was −17.15). This coincides with the Bud Light Boycott of 2023. Calls to action are common in the top terms used when mentioning attention stocks, as shown in Table 6. These include the terms “buy” and “not buy”. Urgent calls to action dominating our findings are similar to other research that suggests the fear of missing out is a mediating device and psychological bias common in retail investing communities [93,94]. The effects of the Bud Light boycott include a strong drop in net sentiment that lasted 13 weeks after they began around April 2^nd^, 2023. It is possible that these figures which aggregate net sentiment of posts about Bud Light are not adequately accounting for the difference between those protesting the advertising campaign, and those disappointed with the company’s reaction to the boycott. As such, we recommend a cautious approach to interpreting these findings. Nevertheless, these effects attenuated and by the end of August 2023, when Bud Light net sentiment returned to its pre-boycott norm of +30.

**Table 6:** Leading Terms for Stocks and Crypto in Social Media Data.

Category	Attention Stocks Leading Terms				Crypto Leading Terms			
	2020	2021	2022	2023	2020	2021	2022	2023
Positive Behavior #1	miss	buy	buy	buy	buy	buy	buy	buy
Positive Behavior #2	buy	miss	miss	drink	use	#shop	use	earn
Negative Behavior #1	boycott	not buy	not buy	boycott	not buy	not buy	not buy	not buy
Negative Behavior #2	#boycott	ban	prohibit	not drink	not use	ban	ban	withdraw
Positive Attribute #1	happy birthday bud	stuff	happy birthday bud	Bud Light	gain	supporter	gain	earn
Positive Attribute #2	great	happy birthday bud	great	happy birthday bud	perfect	gain	long	profit
Negative Attribute #1	yo problem	stock	lose	lose	price	price	lose	devs
Negative Attribute #2	force to close	lose	$AMC short	Bud Light	lose	crash	responsible for loss	price

### 3.4 Crypto

**Fig 7:**
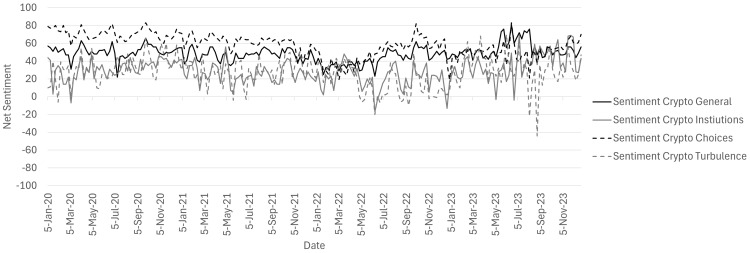
Net Sentiment of Crypto.

**Fig 8:**
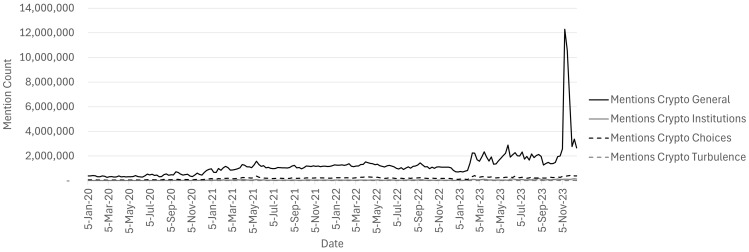
Mentions of Crypto.

Of the four search topic groups (USD, gold, Stocks, and Crypto), Crypto had the most mentions over the data collection period, comprising just over 75% of all recorded. Crypto net sentiment was generally highly positive in 2020–2023, averaging around +50 when evaluated on a weekly basis. This finding is significant as some researchers have found that optimism is a key factor in promoting continued consumer engagement in cryptos [95]. Mentions related to market choices were always the largest of the three sub-searches (compared to sub-searches specific to market *institutions* and market *turbulence*). We visualize Crypto net sentiment in Fig 7 and mention counts in Fig 8.

Crypto rhetoric on social media is largely centered around promotions, calls to action, and influencers using crypto hashtags. Many posts called for consumers to join a group such as a Discord server (where users can message one another), newsletter, or otherwise dedicated investment channel/team for crypto investments. In the context of crypto, hashtags seem to be commonly used to promote other products, including podcasts and personal investment advice, among other products.

Events occurring during our observation period were a mix of good and bad news for crypto markets. Strong growth in 2020–2022, along with increasing acceptance, gave way to legal woes for large crypto trading platforms such as FTX, the leading cryptocurrency asset trading platform, which declared bankruptcy in 2023 [96]. This was followed quickly by law enforcement prosecution of several high-profile criminal cases against leaders of this exchange [97].

While investment decisions by major market players sometimes rose to the top of the discussion, there was a notable lack of discussion in these groups surrounding the collapse of FTX and subsequent criminal investigations. Regarding criminality, the use of cryptocurrencies as a liquidity mechanism to finance criminal activity remains a subject of discussion on social and news media [98].

Despite the challenges faced, crypto has enjoyed a slow march into acceptance by major financial institutions. Notably, this includes the US SEC approving the first bitcoin futures exchange-traded fund (ETF) in October 2021. ETFs, like mutual funds, pool stakeholder money to invest in securities and assets [99]. This gradual acceptance may explain why net sentiment was positive on the institution’s sub-search. The largest jump in mentions in the crypto dataset is observed in early November 2023, consisting of and referring to favorable news coverage of multiple new ETF funds dedicated to cryptocurrencies, including Bitcoin and Ethereum [99]. This was further reinforced by large 2023 increases in average weekly mentions of the sub-search groups on market choices (which saw a 59% increase in average weekly mentions from the year prior) and market institutions (which saw an 82% rise from the year prior). The increase in mentions is substantially greater than that of the market turbulence sub-search (26% over the prior year)

Creating financial instruments out of cryptocurrencies poses a complex series of challenges for institutions, including the banks and the SEC, as cryptocurrencies are not considered securities [99]. Institutions courting cryptocurrencies also pose a dilemma for investors, as increasing levels of institutional interaction will corrode the decentralization that has long been a fixture of cryptocurrencies’ attractiveness [100]. This late jump in mentions (accounting for a nearly 435% rise from the week prior) in the middle of November 2023 did not register strongly in any of the three thematic sub-searches.

Terms frequently used when discussing cryptocurrency are described in Table 6. These closely resemble the stock discussions. Frequent calls to action advertise media platforms (like podcasts or private discussion groups), which promise the opportunity to gain useful information for trading. Commenters also show interest in “earnings” and “profit”, while demonstrating price sensitivity, including the use of the term “loss”.

### 3.5 Test for means and variance

Results of the t-test for means and classical F-test for variance are shown in Table 7. We see that on average, sentiment about attention stocks is lower than sentiment about cryptos by about 9–10 points, indicating a greater number of negative posts about stocks than cryptos. This is intuitive: one of the attention stocks faced a boycott, and although the other two delivered strong returns before the frenzy, their volatility exposed ill-timed investors to significant losses. This contrasts with comparisons of attention stocks and gold as well as attention stocks and USD; both USD and gold see higher mean net sentiment than the selected attention stocks (showing a mean difference of 26.56 and 17.41, respectively). Provided that USD and gold are generally considered safer investments than one of our attention stocks, it is understandable that they would be discussed more favorably than our group of attention stocks are.

**Table 7:** Results of the T-Test for Means and F-Test for Variance in Sentiment.

Comparison (Var1 – Var2)	Mean_1_	Mean_2_	Test Statistic	p-value	Variance test (F Statistic)	Variance Test P Value
Attention Stocks – Crypto	38.75	48.53	−5.34	<0.001	5.67	<0.001
Attention Stocks – Gold	38.75	21.34	9.39	<0.001	2.95	<0.001
Attention Stocks – USD	38.75	12.19	17.26	<0.001	2.63	<0.001
USD – Gold	12.19	21.34	–6.78	<0.001	1.12	0.41
USD – Crypto	12.19	48.53	–29.68	<0.001	2.15	<0.001
Crypto – Gold	48.53	21.34	26.39	<0.001	0.52	<0.001
Crypto Pre/Post Bank Failures	46.87	55.39	−4.82	<0.001	0.57	0.007
Gold Pre/Post Bank Failures	20.62	24.24	−1.43	.157	0.64	0.68
USD Pre/Post Bank Failures	14.72	1.90	4.78	<0.001	0.49	0.68
Attention Stocks Pre/Post Bank Failures	45.42	11.76	7.47	<0.001	0.23	0.68

Comparing mean net sentiments on USD and gold, as well as USD and cryptos, shows that on average, sentiment about USD is lower than gold and Cryptos by 9.15 and 36.34, respectively. Investors departure to gold (even compared to USD) could explain the higher mean net sentiment. This same line of thinking is difficult to reconcile with the higher net sentiment for cryptos. Indeed, we see that cryptos have an even higher mean net sentiment than gold by roughly 21.34, which is significant at the 1% level. Evidently, there is a lot of optimism in crypto markets. This optimism is not surprising, given that some posts suggest an expectation that a cryptocurrency’s price will continue to rise, often using phrases suggesting a rise like “to the moon” [101,102]; phrases like these are sometimes found in alleged pump and dump schemes [103]. In the context of recent literature on the subject, this makes sense; investors in crypto markets tend to behave differently than in more traditional settings [104]. These investors tend to have a greater appetite for risk and higher levels of enthusiasm [105].

The notion that our attention stocks were highly volatile compared to cryptos is reflected in the net sentiment as well. The variance test shows higher levels of variance in stocks, which is significant at the 1% level. This may indicate a wider distribution of opinions on these assets compared to cryptos. Net sentiment of attention stocks also shows a higher variance than gold and USD; both significant at the 1% level. Variances of gold and USD are statistically similar. Comparing the net sentiment of Crypto to USD and gold, we see that crypto has a lower variance than both, with each case significant at the 1% level. Comparing mean net sentiment of the four asset groups before and after the regional bank failures observed around March 10^th^, 2023, cautiously yields some insights. After the bank failures, we see a statistically significant increase in sentiment for cryptos and a statistically significant decrease in net sentiment for the USD and attention stocks. While there is a minor increase in net sentiment of gold after the event, this is not statistically significant and may be noise in the data. Additionally, we cannot attribute causality to any of these events, as they may coincide with other trends or events not directly captured by our sampling strategy.

## 4 Discussion

Across the range of assets studied, we found a consistent theme that public discourse on these subjects is steady, but each market is mutually interdependent with competitors, regulators, and more generally, macroeconomic events. Interest in the USD and gold is largely stable but susceptible to shocks caused by regulatory events, including discussion of a digital dollar and increasing regulatory acceptance of crypto. Previous research has found that investor net sentiment positively influences investment returns [4]. Here, we extend this finding to see that periods of growing regulatory approval of cryptocurrencies are met with increasing investor interest and positive net sentiment. Assets like crypto and attention stocks are subject to mass movement spurred by calls to action on social media. Brand managers should anticipate this as a possibility and consider the long-run strategic impact of these short-run events.

Results of the tests on means and variance are a fruitful basis for discussion in conjunction with recent advances in behavioral finance literature. Social media discussions on investing (specifically prospecting investments) tend to favor stocks with momentum [106]. Based on these findings, it is intuitive that our attention stocks and some cryptos would attract such high amounts of mentions on social media. Further, findings of a recent study of meme-stock traders suggest that those who gather information on social media are likely to have low investment knowledge and may not trade on market fundamentals, instead preferring to trade on sentiment [23,24]. Similar research finds that traders who participate in online investing communities may have a short memory [23].

We see evidence corroborating related findings that social trading (such as through online media) tends to exacerbate the volatility of prices [107], and evidence that our attention stocks are often traded more as bets than genuine investments [108]. As such, it is unsurprising that our attention stocks’ net sentiment shows a higher variance than the other asset groups. In our net sentiment data, crypto also showed relatively high volatility in net sentiment, which is intuitive in relation to recent findings that crypto is an asset that overconfident investors are more likely to buy [109]. It is also intuitive that attention stocks show a higher variance and lower net sentiment than cryptos. Not only are some traders of attention stocks losing money, but recent findings show stronger evidence of rational investors mixed in these markets along with the irrational speculators and those with FOMO, a term to express a fear of missing out (compared to evidence from meme stock markets) [110].

As we have established, assets like gold tend to be viewed as a safe haven. Recent findings suggest that flights to gold are triggered not only in times of high volatility in equity markets, but that medium levels of volatility can trigger these as well [111]. USD and gold exhibit lower variance than crypto and attention stocks, which is intuitive because evidence suggests that these are generally better safe havens than cryptocurrencies are [112]. Provided that our period of observation includes post-COVID economic conditions as well as the Russian invasion of Ukraine, our observations that mentions (and therefore discourse) of gold rise intuitively over the period of observation. This may reflect increased interest in positive and negative connotations.

While mentions of gold rose year by year, we would be remiss to not acknowledge that mentions of cryptos rise by a greater magnitude than gold. It is important to re-acknowledge that our period of observation also aligns with the increasing institutionalization of crypto markets [48,49]. Recent research in behavioral finance suggests that investors may exhibit a salience bias that impacts volatility, specifically that not all risks are weighed evenly by investors [113]. This selective attention found by Polat [113] is likely a driver of interest and volatility in these markets, which is visible in the findings of our F-tests. Institutionalization of crypto assets is likely to have the effect of legitimizing the investment (or at least suppressing other perceived risks associated with them). With Crypto being the market most changing (relative to gold and the USD, which enjoy a long memory of being the safe haven), and stocks (which may be highly volatile), it makes sense that cryptos occupy the middle ground in variance between the safer traditional assets and the more risky attention stocks. Further, F-tests do not show evidence of different volatility between gold and USD. This reinforces the notion that these are both safe assets. The fact that we do not see higher volatility in USD, despite the inflation experienced in the US during the period of observation, suggests that the USD was still viewed as a reliable safe haven during our period of observation.

Examining net sentiment of the asset groups before and after the regional bank collapses that occurred around March 10^th^ 2023, we see increased net sentiment for cryptos after the event, with decreased net sentiment for stocks and USD after the event. We also observe post event variance is higher for each of the asset groups. While this does not establish causality, it does lend some insight into the trends occurring during the period of observation. However, these insights should be interpreted with caution. Sample sizes before and after the event are uneven (with 165 observations before and 41 after), which limits the robustness of the tests.

## 5 Conclusion

Utilizing online and social media data from the US from 2020 to 2023, we gain a better understanding of non-traditional investor community behavior during this time. Using our consistent framework to study assets reflective of their respective classes, we gain a better understanding of the interest and emotional framing specific to each asset group among users of online media. We find high levels of interest in, and enthusiasm for, stocks subject to attention shocks and cryptocurrencies (despite their increasing institutionalization) are frequent topics of conversation. Another common talking point in online and social media are posts reminiscing about the days of the gold standard. Online communities enjoy discussion, debate, market speculation, and calls to action, often in the format of memes or short internet forum comments and posts. Commenters frequently mention opportunities to ‘earn’ and warn one another against ‘missing out’. Many of these participants appear to be retail investors who are excited by the opportunity to punish institutional investor groups and remove themselves from the traditional financial circles and practices of the 21^st^ century. Notably, online media engagement with crypto grew substantially during the data collection period. This growing number of mentions for crypto could have been encouraged by the increasing integration of cryptocurrencies into traditional financial institutions, and there is some evidence of this in top mentions and topics of discussion within the crypto search results.

Many investors rely on financial institutions to permit them to use complex financial options, including short selling. There is no denying that one who played their cards right in these circles could make money, but this may come with enormous risk. Further opportunities for research include surveying individuals to determine investor archetypes in order to compare risk appetites, financial acumen, and market knowledge. Furthermore, the high count of mentions of topics including cryptocurrency and virtual currency in the USD search seems to indicate strong interest in these products among the users of online media studied.

This study is limited in a few ways. Firstly, while online media data is useful to collect high amounts of mentions on a topic, this collection strategy can fall victim to biases in sentiment analysis. To begin with, we did not explicitly exclude bot generated content. As highlighted by Ng and Carley [114] about 20% of global social media chatter stems from bots. While there are inherent linguistic differences between human and bot posts, a key challenge is the systematic identification of bot generated content, an aspect that has become even more challenging with the rise of large language models. We are thus unable to parse out which posts constitute bot and which constitute human posts particularly in the aggregated manner in which data is collected through Quid. Importantly, however, even though bots do not represent real human posts, they can impact the results of sentiment analysis, although the extent to which this is true is a matter of ongoing debate [115,116]. The same is true for echo chambers in online media [117]. It is certainly true that bots can have an effect to amplify certain points of view which may skew sentiment analysis results, filtering these poses a dilemma for this avenue of research. To entirely remove these bot accounts risks, excluding the mechanisms that impact public opinion on the topic of cryptocurrencies and stocks. Future research is ripe for an exploration of the impact of bots and echo chambers and for constructing a better mechanism to account for these in online media without completely negating their impacts.

Opportunities for future research also include a more in-depth study of the drivers of investor interest in cryptocurrencies, and whether institutionalization of these assets into ETFs and mutual funds encourages their adoption. Similarly, this could include an expanded list of assets in each topic to provide a more exhaustive analysis. However, this would likely come at the expense of depth of observation specific to each asset, as the quantity of online media posts on these topics is high. Additionally, future researchers could examine the effect of regional bank failures and adoption of crypto ETFs by using causal inference and quasi-experimental designs.

## Supporting information

S1 File Appendix of tables. (DOCX)

S2 File Minimal dataset for analysis (Excel). (XLSX)

## References

[1] Baer H, Mote L. Banking structures in major countries. Springer Nature; 1992.

[2] Blasco N, Corredor P, Ferreruela S. Market sentiment: a key factor of investors’ imitative behaviour. Accounting & Finance. 2011;52(3):663–89. doi: 10.1111/j.1467-629x.2011.00412.x

[3] Kashyap AK, Stein JC. Monetary Policy When the Central Bank Shapes Financial-Market Sentiment. Journal of Economic Perspectives. 2023;37(1):53–75. doi: 10.1257/jep.37.1.53

[4] Chowdhury EK, Chowdhury R, Dhar BK. Understanding investor sentiment: analyzing its influence on stock and cryptocurrency markets during the Russia–Ukraine war. Thunderbird International Business Review. 2024.

[5] Xu X. Trust and financial inclusion: a cross-country study. Finance Research Letters. 2020;35:101310. doi: 10.1016/j.frl.2019.101310

[6] Fisch JE, Seligman JS. Trust, financial literacy, and financial market participation. Journal of Pension Economics and Finance. 2021;21(4):634–64. doi: 10.1017/s1474747221000226

[7] Adil M, Singh Y, Subhan M, Saleh Al-Faryan MA, Ansari MS. Do trust in financial institution and financial literacy enhances intention to participate in stock market among Indian investors during COVID-19 pandemic?. Cogent Economics & Finance. 2023;11(1). doi: 10.1080/23322039.2023.2169998

[8] Bengoa DS, Kaufmann HR. The influence of trust on the trilogy of knowledge creation, sharing, and transfer. Thunderbird International Business Review. 2015.

[9] Statistica. statistica.com/statistics. 2023. [cited 2024 Sept 18]. https://www.statista.com/statistics/1321847/trust-in-financial-institutions-in-the-us/#:~:text=Trust%20in%20financial%20institutions%20in%20the%20U.S.%202021%20and%202023&text=Less%20people%20had%20trust%20in,nine%20percent%20to%2014%20percent

[10] Zinn A, Neal M, Godinez-Puid L, Perry V. Realestate.business.gwu.edu. 2023. [cited 2024 Sept 18]. https://realestate.business.gwu.edu/sites/g/files/zaxdzs6531/files/2024-04/trust-me-im-a-bank.pdf

[11] NEFE. nefe.org/news. 2023. [cited 2024 Sept 10]. https://www.nefe.org/news/2023/04/opinion-poll-explores-trust-in-financial-institutions.aspx

[12] AP-NORC Center for Public Affairs Research. Few have confidence in financial institutions. AP-NORC Center for Public Affairs Research. 2023. [cited 2024 Sept 10]. https://apnorc.org/projects/few-have-confidence-in-financial-institutions/

[13] Vo LV, Le HTT. From Hero to Zero: The case of Silicon Valley Bank. Journal of Economics and Business. 2023;127:106138. doi: 10.1016/j.jeconbus.2023.106138

[14] Roccograndi H. 2023. [cited 2025 October 9]. https://www.ebsco.com/research-starters/history/collapse-silicon-valley-bank

[15] Aliaga-Diaz R, Hirt J. corporate.vanguard.com/content [Online]. 2024. [cited 2024 Sept 10]. https://corporate.vanguard.com/content/corporatesite/us/en/corp/articles/why-us-dollar-remains-reserve-currency-leader.html#:~:text=Hirt%3A%20There%20are%20three%20primary,U.S.%20government%20and%20its%20institutions

[16] Siripurapu A, Berman N. cfr.org/backgrounder. 2023. [cited 2024 Sept 10]. https://www.cfr.org/backgrounder/dollar-worlds-reserve-currency

[17] JP Morgan. jpmorgan.com/insights [Online]. 2023. [cited 2024 Sept 10]. https://jpmorgan.com/insights/global-research/currencies/de-dollarization

[18] Rodeck D, Dammeyer L. Forbes.com. Forbes. 2024. [cited 2024 Sept 13]. https://www.forbes.com/advisor/investing/guide-to-investing-in-gold/#:~:text=Humans%20have%20used%20gold%20as,assets%20like%20stocks%20and%20bonds

[19] Copeland R, Rhone K. The stock market’s most unserious season is back and dorkier than before. 2025. [cited 2025 Nov 7]. https://www.nytimes.com/2025/07/24/business/opendoor-krispy-kreme-meme-stocks.html

[20] Mark J, Bonos L. Washington Post. 2024. [cited 2025 Nov 7]. https://www.washingtonpost.com/business/2024/12/04/bitcoin-price-100k-crypto-trump/

[21] Hayes A, Scott G. What are meme stocks, and are they real investments? 2025. [cited 2025 Dec 15]. https://www.investopedia.com/meme-stock-5206762

[22] Costola M, Iacopini M, Santagiustina CRMA. On the “mementum” of meme stocks. Economics Letters. 2021;207:110021. doi: 10.1016/j.econlet.2021.110021

[23] Smith ML, Kilders V, Kuethe T, Widmar NO. A time series analysis of herd investor behavior using online and social media data. SAGE Open. 2025;15(3). doi: 10.1177/21582440251375185

[24] Pandey I, Guillemette M. Social media, investment knowledge, and meme stock trading. Journal of Behavioral Finance. 2024;:1–17.

[25] Behl A, Nigam A, Vrontis D. Guest editorial overview: ‘Mapping the future of consumer behaviour using disruptive technologies’. J of Consumer Behaviour. 2024;23(4):1854–8. doi: 10.1002/cb.2309

[26] Brown N, Elliot WB, Wermers R, White R. News or noise: Mobile internet technology and stock market activity. Cologne, De; 2022.

[27] Hirshleifer D, Peng L, Wang Q. News Diffusion in Social Networks and Stock Market Reactions. The Review of Financial Studies. 2024;38(3):883–937. doi: 10.1093/rfs/hhae025

[28] Yamamoto M, Nah S, Bae SY. Social media prosumption and online political participation: an examination of online communication processes. New Media & Society. 2019;22(10):1885–902. doi: 10.1177/1461444819886295

[29] Cheng WH, Ni Y, Ho TH, Chiang CJ, Huang P, Cheng Y. Are the shareholding and trading behaviors of diverse investors affected by the relaxation of day trading?. PLoS One. 2021;16(4). 10.1371/journal.pone.0250121 PMC8064584 33891620

[30] Vaughan M, Gruber JB, Langer AI. The tension between connective action and platformisation: disconnected action in the GameStop short squeeze. New Media & Society. 2023;27(2):632–54. doi: 10.1177/14614448231182617

[31] Martin BAS, Chrysochou P, Strong C. Effects of dispositional greed and need for cognition on. Journal of Consumer Behaviour. 2024;2650–9.

[32] Pablo B. Social media, echo chambers, and political polarization. In: Persily N, Tucker JA, editors. Social Media and Democracy. Cambridge, United Kingdom: Cambridge University Press; 2020. p. 34–55.

[33] Jermann U. NBER. [Online]. 2023. [cited 2024 Sept 17]. https://www.nber.org/papers/w31386

[34] JP Morgan. Currency volatility: Will US dollar strength continue? 2024. [cited 2024 May]. https://www.jpmorgan.com/insights/global-research/currencies/currency-volatility-dollar-strength#:~:text=Despite%20uncertain%20macro%20conditions%2C%20the,other%20major%20currency%20in%202024

[35] Sen J. The weaponisation of the dollar: policy options for small countries. LSE!deas. 2019.

[36] Lahiri U. The Future of Dollar Hegemony. 2023. [cited 2025 Oct 31]. https://www.cfr.org/blog/future-dollar-hegemony

[37] Hall RE. Explorations in the gold standard and related policies for stabilizing the dollar. In: Hall RE, editor. Inflation: causes and effects. Chicago, Illinois: University of Chicago Press; 1982. p. 111–22.

[38] Cutsinger BP. On the feasibility of returning to the gold standard. The Quarterly Review of Economics and Finance. 2020;78:88–97. doi: 10.1016/j.qref.2020.03.002

[39] Cartwright M. worldhistory.org. [Online]. 2014. [cited 2024 May]. https://www.worldhistory.org/gold/

[40] Bordo MB. Macroeconomics, Money and Banking at Econlib. 2023. [cited 2024]. https://www.econlib.org/library/Enc/GoldStandard.html#:~:text=The%20United%20States%2C%20though%20formally,where%20it%20remained%20until%201933

[41] Ghizoni SK. Federal Reserve History. 1971. [cited 2024 May]. https://www.federalreservehistory.org/essays/gold-convertibility-ends

[42] Grand View Research. Cryptocurrency Market (2025 - 2030). [cited 2025 Oct 30]. https://www.grandviewresearch.com/industry-analysis/cryptocurrency-market-report

[43] Griefeld K, Hajric V, Bain B. bloomberg.com/news. 2021. [cited 2024 Aug 10]. https://www.bloomberg.com/news/articles/2021-10-15/bitcoin-futures-etf-said-not-to-face-sec-opposition-at-deadline?embedded-checkout=true

[44] Mejdrich K. Politico.com/news. 2021. [cited 2024 Aug 10]. https://www.politico.com/news/2021/10/19/sec-bitcoin-funds-crypto-516218

[45] Prasad E. The brutal truth about bitcoin. Brookings.edu. 2021. [cited 2024 Sept 10]. https://www.brookings.edu/articles/the-brutal-truth-about-bitcoin/

[46] Greenberg A. 27 year old codebreaker busted myth bitcoins anonymity. Wired. 2024. [cited 2024 Sept 10]. https://www.wired.com/story/27-year-old-codebreaker-busted-myth-bitcoins-anonymity/

[47] Weil D. wsj.com/finance. 2023. [cited 2024 Sept 10]. https://www.wsj.com/finance/currencies/bitcoin-inflation-hedge-84f6b840

[48] Androulaki E, Karame G, Roeschlin M, Scherer T, Capkun S. Bitcoin, evaluating user privacy in bitcoin. Cryptography and Data. 2023.

[49] Chow A. time.com. 2022. [cited 2024 Sept 10]. https://time.com/6239364/crypto-criminals-andy-greenberg/

[50] Davidson J. Money.com. 2018. [cited 2024 May]. https://money.com/wall-street-bets/

[51] Stewart E. vox.com. 2024. [cited 2024]. https://www.vox.com/the-goods/22249458/gamestop-stock-wallstreetbets-reddit-citron

[52] Investor.gov. investor.gov/introduction-investing. 2024. [cited 2024 Sept 10]. https://www.investor.gov/introduction-investing/investing-basics/how-stock-markets-work/stock-purchases-and-sales-long-and

[53] Steiner R, Clark A. Barrons.com/articles. 2024. [cited 2024 May]. https://www.barrons.com/articles/gamestop-amc-stock-price-meme-roaring-kitty-621ad9c1

[54] Duarte F. Explodingtopics.com. 2024. [cited 2024 June]. https://explodingtopics.com/blog/video-streaming-stats

[55] Desiderio A, Aiello LM, Cimini G, Alessandretti L. The dynamics of the Reddit collective action leading to the GameStop short squeeze. npj Complex. 2025;2(1). doi: 10.1038/s44260-025-00029-z

[56] Securities and Exchange Commission US. sec.gov. 2021. [cited 2024 May]. https://www.sec.gov/news/public-statement/joint-statement-market-volatility-2021-01-29?utm_medium=email&utm_source=govdelivery

[57] Reuters. Reuters.com/world. Reuters. 2021. [cited 2024 Sept 10]. https://www.reuters.com/world/us/us-sec-says-observing-market-meme-stocks-rally-2021-06-07/

[58] Baresa S, Bogdan S, Ivanovic Z. Strategy of stock valuation by fundamental analysis. UTMS Journal of Economics. 2012;:44–51.

[59] Kim K, Lee S-YT, Kauffman RJ. Social informedness and investor sentiment in the GameStop short squeeze. Electron Mark. 2023;33(1):23. doi: 10.1007/s12525-023-00632-9 37252673 PMC10203679

[60] Reuters. Can WallStreetBets’ spectacular short squeeze be repeated?. 2022. [cited 2024 October]. https://www.reuters.com/plus/can-wallstreetbets-spectacular-short-squeeze-be-repeated

[61] Federal Reserve Bank of Richmond. Federal Reserve Bank of Richmond. fraser.stlouisfed.org. 2021. [cited 2024 May]. https://fraser.stlouisfed.org/files/docs/historical/frbrich/econbrief/frbrich_eb_21-13.pdf

[62] Hansen AL, Lee SJ. Financial stability implications of generative AI: taming the animal spirits. Finance and Economics Discussion Series. Federal Reserve Board; 2025.

[63] Fisk AE, Smith ML, Richert BT, Olynk Widmar NJ. #PorkandPigs: an online media listening analysis of public perception of the U.S. swine industry. Transl Anim Sci. 2024;8:txae155. doi: 10.1093/tas/txae155 39660285 PMC11630858

[64] Myers O. The Guardian. 2023. [cited 2024 June]. https://www.theguardian.com/world/2023/sep/19/dylan-mulvaney-bud-light-boycott

[65] Villagra N, Monfort A, Méndez-Suárez M. Firm value impact of corporate activism: Facebook and the stop hate for profit campaign. Journal of Business Research. 2021;137:319–26. doi: 10.1016/j.jbusres.2021.08.052

[66] Lai J, Bir C, Widmar NO. Public sentiment towards cruises and resulting stock performance in 2017–2021. Journal of Hospitality and Tourism Management. 2023;56:1–7. doi: 10.1016/j.jhtm.2023.05.011

[67] Jung J, Widmar NO, Subramani S, Feng Y. Online media attention devoted to flour and flour-related food safety in 2017 to 2020. J Food Prot. 2022;85(1):73–84. doi: 10.4315/JFP-21-085 34347869 PMC9906424

[68] Smith ML, Berenda AB, Kilders V, Widmar NO, Bir C, Bailey NF. Social media perceptions of college football performance and season length 2019–2023. PLoS One. 2025. 10.1371/journal.pone.0325840 PMC12212522 40591618

[69] Quid. Quid Knowledge Hub. 2023. [cited 2024 May] https://www.quid.com/knowledge-hub/resource-library/blog/natural-language-processing-guide/

[70] Bir C, Widmar NO, Clifford M. The Intersection of “Natural” Edutainment and Perceptions of Natural Resource Uses. Environmental Communication. 2019;14(2):168–83. doi: 10.1080/17524032.2019.1601634

[71] Widmar NO, Bir C, Clifford M, Slipchenko N. Social media sentimentas an additional performance measure? Examples from iconic theme park destinations. Journal of Retailing and Consumer Services. 2020;56:102157. doi: 10.1016/j.jretconser.2020.102157

[72] Quid. Quid security policy. [cited 2023] https://www.quid.com/hubfs/NetBase_Quid_security_policy%20Final%2010.4.23.pdf

[73] Quid. [cited 2024]. https://www.quid.com/privacy

[74] Duggan W. Money.usnews.com. 2024. [cited 2024 May]. https://money.usnews.com/investing/cryptocurrency/articles/bitcoin-vs-ethereum-which-is-a-better-buy

[75] Stata. stata.com/manuals. [Online]; 2025. chrome-extension://efaidnbmnnnibpcajpcglclefindmkaj/https://www.stata.com/manuals/rttest.pdf

[76] NIST. Itl.nist.gov. 2012. [cited 2023]. https://www.itl.nist.gov/div898/handbook/eda/section3/eda359.htm

[77] Xe.com. [cited 2024]. https://xe.com/currencycharts/?from=USD&to=EUR

[78] Library of Congress. Congress.gov. [cited 2024]. https://www.congress.gov/bill/118th-congress/house-bill/2435

[79] Legiscan. Texas House Bill 4903. [Online]. 2023. https://legiscan.com/TX/text/HB4903/id/2803474

[80] Michel N. Cato.org. [Online]. 2023. [cited 2024 Sept 13]. https://www.cato.org/testimony/digital-dollar-dilemma-implications-central-bank-digital-currency-private-sector

[81] Faulkender M, Vasquez D. America First Policy Initiative. [cited 2024 Sept 13]. https://americafirstpolicy.com/issues/research-report-central-bank-digital-currencies

[82] Schroeder P. Reuters. 2023. [cited 2024 Sept 13]. https://www.reuters.com/markets/us/feds-barr-says-central-bank-a-long-way-any-decision-issuing-digital-currency-2023-09-08/

[83] Bernstein J. The July Consumer Price Index: It’s All About That Base (Effect). 2023. [cited 2024 May.]. https://www.whitehouse.gov/cea/written-materials/2023/08/10/the-july-consumer-price-index-its-all-about-that-base-effect/#:~:text=Consumer%20prices%2C%20as%20measured%20by,higher%20than%20June’s%203%20percent

[84] CNBC. CNBC.com. 2023. [cited 2024 Aug]. https://www.cnbc.com/2023/03/13/safe-haven-gold-accelerates-as-traders-assess-svb-fallout.html#:~:text=Gold%20and%20silver%20prices%20surged,on%20its%20aggressive%20monetary%20policy

[85] Carballo R. Customers flock to Costco to buy gold bars. 2024. [cited 2024 May]. https://www.nytimes.com/2024/04/11/business/costco-gold-bars.html

[86] Sunderaraman P, Ho S, Chapman S, Joyce JL, Colvin L, Omollo S, et al. Technology use in everyday financial activities: evidence from online and offline survey data. Arch Clin Neuropsychol. 2020;35(4):385–400. doi: 10.1093/arclin/acz042 31696205 PMC7244884

[87] Ryan M, Corbet S, Oxley L. Is gold always a safe haven?. Finance Research Letters. 2024.

[88] Vasileiou E, Tzanakis P. The Impact of Google Searches, Put-Call Ratio, and Trading Volume on Stock Performance using wavelet coherence analysis: the AMC case. Journal of Behavioral Finance. 2022;25(1):111–9. doi: 10.1080/15427560.2022.2100384

[89] Pittman T. AMC reopens theaters, offers 15-cent tickets on Aug. 20. 2020. [cited 2024 May]. https://www.wthr.com/article/news/health/coronavirus/amc-theaters-reopen-august-20/507-9779a5c9-b961-48f2-9a41-ec0e7b73e01f

[90] Tassi P. What GameStop is doing in the coronavirus pandemic is inexcusable. 2020. [cited 2024 May]. https://www.forbes.com/sites/paultassi/2020/03/20/what-gamestop-is-doing-in-the-coronavirus-pandemic-is-inexcusable/?sh=45e3d0b17036

[91] Graham J. usatoday.com. 2022. [cited 2024 Aug]. https://www.usatoday.com/story/tech/2020/03/22/gamestop-after-backlash-closes-retail-doors-due-covid-19-concerns/2894745001/

[92] Gross J. The New York Times. 2020. [cited 2024 May]. https://www.nytimes.com/2020/06/18/business/AMC-theaters-masks-coronavirus.html

[93] Ivantchev B, Ivantcheva M. FOMO effect: social media and online traders. JMFS. 2024;(48). doi: 10.33119/jmfs.2023.48.4

[94] Nani A. All are investing in crypto, I fear of being missed out: examining the influence of herding, loss aversion, and overconfidence in the cryptocurrency market with the mediating effect of FOMO. Quality & Quantity. 2025:2237–63.

[95] Ahsan ZB, Gupta A. Gamified cryptocurrency and rewards: Consumer behaviour during uncertain earnings. J of Consumer Behaviour. 2023;23(3):1540–55. doi: 10.1002/cb.2291

[96] Pinkerton J, Divine J. Money.usnews.com. 2024. [cited 2024 May]. https://money.usnews.com/investing/articles/the-history-of-bitcoin

[97] US Securities and Exchange Commission. sec.gov. 2022. [cited 2024 May]. https://sec.gov

[98] Varun V. Prospects and models of combating cryptocurrency crimes. Eurocrim. 2024;:365–70.

[99] Nandini E. Forbes. 2023. [cited 2024 May]. https://www.forbes.com/sites/digital-assets/2023/11/09/blackrocks-ethereum-etf-filing-has-big-implications-for-investors/

[100] Ward SV. Bitcoin’s crossroads with spot bitcoin ETF: Decentralization vs. institutionalization. Forbes. 2023. [cited 2024 May]. https://www.forbes.com/sites/digital-assets/2023/11/10/bitcoins-crossroads-with-spot-bitcoin-etf-decentralization-vs-institutionalization/

[101] Peterson T. To the moon: a history of bitcoin price manipulation. Journal of Forensic and Investigative Accounting. 2022;13(2).

[102] Kovacs S. Are investors really moonstruck? Lunar phases, returns, and volatility in global equities and cryptocurrencies. In: SSRN, 2025.

[103] Kamps J, Kleinberg B. To the moon: defining and detecting cryptocurrency pump-and-dumps. Crime Sci. 2018;7(1). doi: 10.1186/s40163-018-0093-5

[104] Kogan S, Makarov I, Niessner M, Schoar A. Are cryptos different? Evidence from retail trading. Journal of Financial Economics. 2024;159:103897. doi: 10.1016/j.jfineco.2024.103897

[105] Weber M, Candia B, Coibion O, Gorodnichenko Y. Do you even crypto, bro? Cryptocurrencies in household finance. 31284. NBER; 2023.

[106] Reichenbach F, Walther M. Attention allocation of investors on social media: the role of prospect theory. Journal of Behavioral Finance. 2024;26(3):317–34. doi: 10.1080/15427560.2024.2309145

[107] Aloosh A, Ouzan S, Shahzad SJH. Bubbles across meme stocks and cryptocurrencies. Finance Research Letters. 2022;49:103155. doi: 10.1016/j.frl.2022.103155

[108] Philander KS. Meme asset wagering: perceptions of risk, overconfidence, and gambling problems. Addict Behav. 2023;137:107532. doi: 10.1016/j.addbeh.2022.107532 36332515

[109] Chhatwani M, Parija AK. Who invests in cryptocurrency? The role of overconfidence among American investors. Journal of Behavioral and Experimental Economics. 2023;107:102107. doi: 10.1016/j.socec.2023.102107

[110] Güler D. The impact of investor sentiment on bitcoin returns and conditional volatilities during the era of covid-19. Journal of Behavioral Finance. 2023;:276–89.

[111] Bouri E, Jalkh N. Flight-to-safety across time and market conditions. International Review of Economics & Finance. 2024;94:103363. doi: 10.1016/j.iref.2024.05.042

[112] Feder-Sempach E, Szczepocki P, Bogołębska J. Global uncertainty and potential shelters: gold, bitcoin, and currencies as weak and strong safe havens for main world stock markets. Financ Innov. 2024;10(1). doi: 10.1186/s40854-023-00589-w

[113] Polat AY. Investor bias, risk and price volatility. JES. 2022;50(7):1317–35. doi: 10.1108/jes-04-2022-0211

[114] Ng LHX, Carley KM. A global comparison of social media bot and human characteristics. Sci Rep. 2025;15(1):10973. doi: 10.1038/s41598-025-96372-1 40164745 PMC11958817

[115] Hartmann D, Wang SM, Pohlmann L, Berendt B. A systematic review of echo chamber research: comparative analysis of conceptualizations, operationalizations, and varying outcomes. J Comput Soc Sc. 2025;8(2). doi: 10.1007/s42001-025-00381-z

[116] Broto Cervera R, Pérez-Solà C, Batlle A. Overview of the Twitter conversation around #14F 2021 Catalonia regional election: an analysis of echo chambers and presence of social bots. Soc Netw Anal Min. 2024;14(1). doi: 10.1007/s13278-024-01251-8

[117] Jiang J, Ren Z, Ferrara E. Social media polarization and echo chambers in the context of COVID-19: case study. JMIRx Med. 2021;:2, 3. 10.2196/29570 PMC8371575 34459833

