# Supplementary material for: To the moon: Retail investor attention and sentiment across asset types in online media
Source: PLoS One. 2026 Jun 10;21(6):e0349616. doi: 10.1371/journal.pone.0349616 (PMC13252755; doi:10.1371/journal.pone.0349616)
Supplement: S1 File — (DOCX) [file pone.0349616.s001.docx]

Appendix I: Mention Data

| **Topical Search** | **Count of Total Mentions** | **% of Mentions** |
| --- | --- | --- |
| USD | 41,611,383 | 11.82% |
| Gold | 1,991,283 | 0.57% |
| Stocks | 41,988,310 | 11.93% |
| Crypto | 266,398,091 | 75.68% |
| Total | 351,989,067 | 100.00% |

Appendix II: Descriptive Statistics

AP2.1 Four-year count of topical search and sub-search mentions showing in-group breakdowns.

| **Values** | **Mention Count** | **% of Group Total** |
| --- | --- | --- |
| Sum of Mentions Total USD | 41,611,383 | 100.00% |
| Sum of Mentions Institutions USD | 5,137,945 | 12.35% |
| Sum of Mentions Market Choices USD | 9,130,085 | 21.94% |
| Sum of Mentions Market Turbulence USD | 1,945,546 | 4.68% |
| Sum of Mentions Crypto Total | 266,398,091 | 100.00% |
| Sum of Mentions Crypto Institutions | 10,620,236 | 3.99% |
| Sum of Mentions Crypto Choices | 43,495,898 | 16.33% |
| Sum of Mentions Crypto Turbulence | 2,190,789 | 0.82% |
| Sum of Mentions Crypto Total (ETH) | 119,160,126 | 44.73% |
| Sum of Mentions Crypto Total (BTC) | 173,010,890 | 64.94% |
| Sum of Mentions Gold Total | 1,991,283 | 100.00% |
| Sum of Mentions Gold Institutions | 1,301,995 | 65.38% |
| Sum of Mentions Gold Choices | 314,298 | 15.78% |
| Sum of Mentions Gold Turbulence | 200,418 | 10.06% |
| Sum of Mentions Stocks Total | 41,988,310 | 100.00% |
| Sum of Mentions Stocks Institutions | 1,320,052 | 3.14% |
| Sum of Mentions Stocks Choices | 6,382,878 | 15.20% |
| Sum of Mentions Stocks Turbulence | 321,699 | 0.77% |
| Sum of Mentions Stocks Total (AMC) | 13,698,952 | 32.63% |
| Sum of Mentions Stocks Total (Bud Light) | 21,830,995 | 51.99% |
| Sum of Mentions Stocks Total (GameStop) | 8,683,323 | 20.68% |

Note: Specific assets searched (ETH. BTC, AMC, GME, BUD) figures show % of group total dataset.

AP2.2: Descriptive Statistics of the USD Social Media Data.

| **USD Total** | | | | |
| --- | --- | --- | --- | --- |
|  | **2020** | **2021** | **2022** | **2023** |
| Average Sentiment | 12.28 | 17.74 | 14.55 | 3.58 |
| (St. Dev) | (8.92) | (11.04) | (12.84) | (16.13) |
| Average Mentions | 108,097 | 249,566 | 224,557 | 210,649 |
| (St. Dev) | (16,587) | (70,776) | (43,141 ) | (58,964) |
| **USD Market Turbulence** | | | | |
|  | **2020** | **2021** | **2022** | **2023** |
| Average Sentiment  (St. Dev) | -20.21 | -12.89 | -15.60  (14.22) | -14.11  (13.32) |
|  | (16.29) | (16.36) |  |  |
| Average Mentions  (St. Dev) | 3,755 | 6,838 | 13,292 | 13,033 |
|  | (894) | (2,069) | (4,062) | (3,624) |
| **USD Market Choices** | | | | |
|  | **2020** | **2021** | **2022** | **2023** |
| Average Sentiment  (St. Dev) | 15.36 | 23.28 | 21.94 | 25.32 |
|  | (9.63) | (11.41) | (14.42) | (13.36) |
| Average Mentions  (St. Dev) | 23,673 | 58,596 | 59,025 | 32,777 |
|  | (4,421) | (11,680) | (12,954) | (6,672) |
| **USD Institutions** | | | | |
|  | **2020** | **2021** | **2022** | **2023** |
| Average Sentiment  (St. Dev) | -3.62 | -2.75 | -6.09 | -12.55 |
|  | (11.85) | (12.48) | (10.55) | (14.34) |
| Average Mentions  (St. Dev) | 13,293 | 23,502 | 29,828 | 31,029 |
|  | (2,944) | (4,041) | (5,497) | (9,862) |
|  | | | |  |

Note: The change in geolocation classification allowed increased location identification which may drive part of the shift in mentions in 2023.

AP2.3: Descriptive Statistics of the Gold Social Media Data.

| **Gold Total** | | | | | |
| --- | --- | --- | --- | --- | --- |
|  | **2020** | **2021** | **2022** | **2023** | |
| Average Sentiment  (St. Dev) | 17.7 | 21.57 | 20.96 | 23.53 | |
|  | (14.22) | (12.15) | (10.48) | (13.52) | |
| Average Mentions  (St. Dev) | 8,660 | 8,465 | 9,223 | 11,690 | |
|  | (2,894) | (1,652) | (2,169) | (3,690) | |
| **Market Turbulence** | | | | | |
|  | **2020** | **2021** | **2022** | **2023** | |
| Average Sentiment  (St. Dev) | -11.96 | -6.26 | 4.89 | 0.34 | |
|  | (22.62) | (15.34) | (17.15) | (18.88) | |
| Average Mentions  (St. Dev) | 584 | 792 | 1,136 | 1,317 | |
|  | (355) | (320) | (295) | (501) | |
| **Market Choices** | | | | | |
|  | **2020** | **2021** | **2022** | **2023** | |
| Average Sentiment  (St. Dev) | 7.79 | 14.79 | 8.79 | 6.74 |  |
|  | (21.33) | (17.88) | (18.52) | (17.39) |  |
| Average Mentions  (St. Dev) | 1,181 | 1,425 | 1,736 | 1,650 |  |
|  | (444) | (418) | (956) | (503) |  |
| **Institutions** | | | | | |
|  | **2020** | **2021** | **2022** | **2023** | |
| Average Sentiment  (St. Dev) | 35.23 | 30.4 | 32.34 | 34.23 | |
|  | (16.31) | (15.17) | (11.01) | (16.27) | |
| Average Mentions  (St. Dev) | 5,881 | 5,272 | 5,723 | 7,985 | |
|  | (2,103) | (938) | (1,008) | (2,679) | |

Note: The change in geolocation classification allowed increased location identification which may drive part of the shift in mentions in 2023.

AP2.4: Descriptive Statistics for Meme Stock Social Media Data.

| **Meme Stock Total** | | | | |
| --- | --- | --- | --- | --- |
|  | **2020** | **2021** | **2022** | **2023** |
| Average Sentiment  (St. Dev) | 43.42 | 46.96 | 46.28 | 18.7 |
|  | (18.26) | (9.89) | (10.88) | (28.05) |
| Average Mentions  (St. Dev) | 100,780 | 209,224 | 151,351 | 336,167 |
|  | (19,965) | (265,743) | (25,282) | (179,722) |
| **Meme Stock Market Turbulence** | | | | |
|  | **2020** | **2021** | **2022** | **2023** |
| Average Sentiment  (St. Dev) | 20.11 | 14.72 | 34.58 | 3.28 |
|  | (36.15) | (35.21) | (32.14) | (40.54) |
| Average Mentions  (St. Dev) | 338 | 1,190 | 2,613 | 1,948 |
|  | (189) | (1,076) | (5,151) | (1,732) |
| **Meme Stock Market Choices** | | | | |
|  | **2020** | **2021** | **2022** | **2023** |
| Average Sentiment  (St. Dev) | 30.45 | 35 | 35.43 | 17.11 |
|  | (14.5) | (11.13) | (15.66) | (24.22) |
| Average Mentions  (St. Dev) | 6,287 | 48,523 | 26,843 | 39,352 |
|  | (2,316) | (96,089) | (9,778) | (19,413) |
| **Meme Stock Institutions** | | | | |
|  | **2020** | **2021** | **2022** | **2023** |
| Average Sentiment  (St. Dev) | 27.4 | 14.47 | 10.77 | -17.15 |
|  | (34.1) | (25.87) | (22.09) | (38.28) |
| Average Mentions  (St. Dev) | 1,229 | 7,631 | 5,886 | 10,252 |
|  | (1,445) | (12,585) | (3,288) | (7,694) |
|  |  |  |  |  |

Note: The change in geolocation classification allowed increased location identification which may drive part of the shift in mentions in 2023.

AP2.5: Descriptive Statistics for Crypto Search

| **Crypto Total** | | | | |
| --- | --- | --- | --- | --- |
|  | **2020** | **2021** | **2022** | **2023** |
| Average Sentiment  (St. Dev) | 50.74 | 47.25 | 42.43 | 54.17 |
|  | (6.94) | (6.13) | (8.67) | (9.9) |
| Average Mentions  (St. Dev) | 425,584 | 1,080,304 | 1,174,164 | 2,388,470 |
|  | (124,704) | (151,254) | (152,697) | (2,215,756) |
| **Crypto Market Turbulence** | | | | |
|  | **2020** | **2021** | **2022** | **2023** |
| Average Sentiment  (St. Dev) | 32.3 | 29.45 | 15.62 | 31.26 |
|  | (15.16) | (12.95) | (16.92) | (19.33) |
| Average Mentions  (St. Dev) | 3,427 | 9,476 | 12,774 | 15,953 |
|  | (1,448) | (3,496) | (4,248) | (6,742) |
| **Crypto Market Choices** | | | | |
|  | **2020** | **2021** | **2022** | **2023** |
| Average Sentiment  (St. Dev) | 71.25 | 61.42 | 49.09 | 51.36 |
|  | (6.09) | (7.58) | (14.17) | (10.87) |
| Average Mentions  (St. Dev) | 70,498 | 202,124 | 216,595 | 338,421 |
|  | (19,757) | (40,334) | (40,655) | (538,839) |
| **Crypto Institutions** | | | | |
|  | **2020** | **2021** | **2022** | **2023** |
| Average Sentiment  (St. Dev) | 31.17 | 26.49 | 20.32 | 36.06 |
|  | (10.88) | (10.44) | (13.36) | (16.22) |
| Average Mentions  (St. Dev) | 17,799 | 50,623 | 47,823 | 85,662 |
|  | (5,913) | (14,580) | (10,420) | (37,034) |

Note: The change in geolocation classification allowed increased location identification which may drive part of the shift in mentions in 2023.

AP2.6: Descriptive Statistics for Individual Assets Social Media Data.

| **Bitcoin** | | | | |
| --- | --- | --- | --- | --- |
|  | **2020** | **2021** | **2022** | **2023** |
| Average Sentiment  (St. Dev) | 51.71 | 48.12 | 40.92 | 46.85 |
|  | (7.5) | (6.99) | (9.29) | (8.5) |
| Average Mentions  (St. Dev) | 407,689 | 957,429 | 868,127 | 1,080,197 |
|  | (133,908) | (138,620) | (119,802) | (623,231) |
| **Ethereum** | | | | |
|  | **2020** | **2021** | **2022** | **2023** |
| Average Sentiment  (St. Dev) | 75.33 | 69.69 | 67.94 | 65.42 |
|  | (7.93) | (4.47) | (7.25) | (12.89) |
| Average Mentions  (St. Dev) | 84,356 | 265,453 | 452,259 | 1,462,724 |
|  | (23,074) | (79,706) | (68,289) | (1,922,277) |
| **GameStop** | | | | |
|  | **2020** | **2021** | **2022** | **2023** |
| Average Sentiment  (St. Dev) | 8 | 21.81 | 26.34 | 25.28 |
|  | (27.67) | (12.84) | (16.43) | (14.3) |
| Average Mentions  (St. Dev) | 16,320 | 79,332 | 32,727 | 36,268 |
|  | (14,679) | (229,107) | (11,715) | (20,131) |
| **Bud Light** | | | | |
|  | **2020** | **2021** | **2022** | **2023** |
| Average Sentiment  (St. Dev) | 57.63 | 64.52 | 62.08 | 19.04 |
|  | (13.17) | (8.81) | (5.56) | (35.85) |
| Average Mentions  (St. Dev) | 70,506 | 64,619 | 68,511 | 215,748 |
|  | (7,752) | (7,831) | (13,339) | (165,496) |
| **AMC** | | | | |
|  | **2020** | **2021** | **2022** | **2023** |
| Average Sentiment  (St. Dev) | 19 | 37.77 | 27.62 | 31.44 |
|  | (27.2) | (12) | (16.06) | (15.82) |
| Average Mentions  (St. Dev) | 15,243 | 88,784 | 60,349 | 97,996 |
|  | (9,140) | (99,335) | (15,855) | (35,115) |

Note: the Bud Light, GameStop and AMC data is a subset of the Meme-Stocks Dataset, and the Bitcoin and Ethereum data are a subset of the Crypto dataset.

Appendix III: Excluded Terms

Crypto excluded terms: church, Sweatcoin, sweatcoin, merch, Merch, #merch, Digital Asset News, #americasuntoldstories, America's Untold Stories, JUST CLAIMED $670 WORTH, Buycrackcocaineonlinecana, healthyhair, vegan, CHURCH, @discoverministries, discoverministries, Steve Cioccolanti, airdrop, Huffman_ETH98, walletcollect99, Airdrop, #airdrop, #Airdrop, affiliate link, Affiliate link, Affiliate Link, $LOYAL, $PSYOP, $BEN, $PEPE, Sign up, Tag 3 Friends, Official URL, email address, FTX Token

Gold Excluded Terms:

Lakers, Kobe, Kobe Bryant, Simone Gold, Lamestream Media, Lamestream Media Gold, RexChapman, Bitch, Fool's gold, fool's Gold, GOLD STANDARD COVID-19 TEST, GOLD STANDARD TEST FOR COVID19, GOLD STANDARD TEST FOR COVID-19, Presidential Daily Briefing, presidential daily briefing, threats to American troops, threats against American troops, public health, Gold Standard of Education, Genocidal Covid Dictators, Osama Bin Laden, caged children, separated families, cat's hind legs, essential liberties post-9/11, polio vaccine & many achievements, advanced medicine & saved lives, modern medicine, Optimum Nutrition Gold Standard, Absolute Gold Standard, PCR test, Chain of Custody, All-Star game, MLB decision, FDA and CDC, control COVID, meta analysis, Supreme Court, Andrew Cuomo, COVID-19 leadership, only 7 deaths, air pods, airpods, Meryl Streep, hetero romance, Amy Coney Barrett, American Bar Association, Chuck Todd, @drdavidsamadi, @RealJamesWoods, @ilyseh, gold standard for HIRING journalists, Lysol Laundry Sanitizer, Star Wars: Clone Wars, Hemodynamic, Star Wars, Pulmonary, FDA approval, Lou Mongello, Cuomo’s leadership during the pandemic, sexual harassing, Surgeon General, Cuomo's, Cuomo, Cuomo:, mRNA, vaccine, vaccines, hypercapnia, Disney podcasts, N95 mask, Peyton Manning, Randomized control trials, placebo-controlled, 000 donation, Nico Collins, $25000 donation, mail-in voting, Qrazies, Gold Glove, gold glove, golden glov, golden glove, Golden glove, Golden Glove, Golden Globe, golden globe, gold standard of research, gold standard of football, gold standard of vaccines, gold standard of movies, gold standard of policy, gold standard of tv, gold standard of care, gold standard of pain assessment, gold standard of medical research, retinoids, DeepMind, gold standard test, gold standard of assessment, gold standard for surface-to-air-missile defense, gold standard of corroboration, JD Vance, gold standard of senate candidates, gold standard of Republican Senate candidates, gold-standard for every governor, Kyle Rittenhouse, N95, surgical masks, @celtics, Roe vs. Wade, autoimmune, methotrexate, gold standard for, gold standard of, @TheWrightCards, Martha's Vineyard, Marthas Vineyard, Lindell, Mike Lindell, comma's importance, Jack Smith, ICU attending, surgical attending, gold standard in justices, gold standard in Justices, Clarence Thomas, Thomas and Alito, Washington Redskins, LEED gold, LEED Gold, LEED, HVAC, AC, A/C

Stocks Excluded Terms:

sports bra, air bud, airbud, shortHairyMeat, @shortHairyMeat, @Bud_Doggin, tastebuds, taste buds, buddy, gardening, flowering, flowers, earbud, ear bud, ear buds, tiddies, cats, nip that in the bud, nip in the bud, nip it in the bud, nip, foodii, FOODii, munched on, Burr oaks, live oaks, Burr Oaks, Live Oaks, Thomas James - Investing, moomoo, Get 13 Free, Get 13 FREE, ThomasJamesPropertyInvesting, @ThomasJamesPropertyInvesting, https://thomasjamesinvesting.com, Red Buds, monster energy, Bud's Gun Shop, Doctor Strange, K9 bud, K9 Bud, airdrop, air drop, Brian Kemp, Kemp, ellipsoidal yeast cells, Bud Grant, Bud Grant passed away, Likith kella, @MalkeAsaad, @benpsyopvip, $PSYOP, AMC exams, @manikmadaan, ok bud, Sorry bud, @bud_cann

USD Excluded Terms:

onlyfans, Only Fans, only fans, airdrop, #airdrops, airdrops, BTC, tokens, Tokens, #crypto, haircut and beard trim, sons of bitches, free gift, Diamonds Will Control Your Life, Bitball merchandise, Bitball, users' unique treasure items, users' unique, Diamonds Will, diamonds will control your life
